# Dosimetric verification for intensity-modulated arc therapy plans by use of 2D diode array, radiochromic film and radiosensitive polymer gel

**DOI:** 10.1093/jrr/rrt139

**Published:** 2014-01-20

**Authors:** Naoki Hayashi, Ryan L. Malmin, Yoichi Watanabe

**Affiliations:** 1 Faculty of Radiological Technology, School of Health Sciences, Fujita Health University, 1-98, Dengakugakubo, Kutsukake-cho, Toyoake, Aichi, 470-1192, Japan; 2 Ridgeview Regional Radiation Oncology, 560 South Maple Street, Suite 10, Waconia, MN 55387, USA; 3 Department of Radiation Oncology, 420 Delaware St. SE, MMC494, Minneapolis, MN 55455, USA

**Keywords:** IMAT, dosimetric verification, 3D dose measurement, radiosensitive polymer gel, radiochromic films, 2D array detector

## Abstract

Several tools are used for the dosimetric verification of intensity-modulated arc therapy (IMAT) treatment delivery. However, limited information is available for composite on-line evaluation of these tools. The purpose of this study was to evaluate the dosimetric verification of IMAT treatment plans using a 2D diode array detector (2D array), radiochromic film (RCF) and radiosensitive polymer gel dosimeter (RPGD). The specific verification plans were created for IMAT for two prostate cancer patients by use of the clinical treatment plans. Accordingly, the IMAT deliveries were performed with the 2D array on a gantry-mounting device, RCF in a cylindrical acrylic phantom, and the RPGD in two cylindrical phantoms. After the irradiation, the planar dose distributions from the 2D array and the RCFs, and the 3D dose distributions from the RPGD measurements were compared with the calculated dose distributions using the gamma analysis method (3% dose difference and 3-mm distance-to-agreement criterion), dose-dependent dose difference diagrams, dose difference histograms, and isodose distributions. The gamma passing rates of 2D array, RCFs and RPGD for one patient were 99.5%, 96.5% and 93.7%, respectively; the corresponding values for the second patient were 97.5%, 92.6% and 92.9%. Mean percentage differences between the RPGD measured and calculated doses in 3D volumes containing PTVs were –0.29 ± 7.1% and 0.97 ± 7.6% for the two patients, respectively. In conclusion, IMAT prostate plans can be delivered with high accuracy, although the 3D measurements indicated less satisfactory agreement with the treatment plans, mainly due to the dosimetric inaccuracy in low-dose regions of the RPGD measurements.

## INTRODUCTION

Radiation therapy technology is rapidly evolving. The recent introduction of the intensity-modulated rotational delivery technique “intensity-modulated arc therapy” (IMAT), and volumetric-modulated arc therapy (VMAT), into radiation oncology clinics expands our armaments for precise delivery of radiation to a target [1]. One of the distinctive advantages of IMAT over intensity-modulated radiation therapy (IMRT) is the potentially faster treatment delivery without degrading the dosimetric superiority of IMRT relative to the more conventional 3D conformal radiation therapy [2]. The IMAT technology, however, is technically more complex than IMRT, because the dose rate changes and the leaves of the multileaf collimator (MLC) move dynamically while the gantry rotates. Hence, more stringent verification and quality assurance programs than those for IMRT are required to introduce the IMAT technology into routine clinical use [3]. A key difference between the new form of IMAT and previous technologies such as Tomotherapy^®^ (Accuray, Sunnyvale, CA) is that IMAT uses a cone-beam delivery technique as opposed to the narrow slice-like field of the earlier technologies.

For dosimetric verification of the IMAT delivery technique, new dosimetric tools have been developed and being marketed [4, 5]. These include, e.g. ArcCheck^®^ from Sun Nuclear Co. (Melbourne, FL) and Delta4^®^ from ScandiDos AB (Uppsala, Sweden). These devices incorporate thousands of diode detectors placed on a cylindrical surface or on two orthogonal planes. Other devices that use arrays of diodes or ionization chambers include MapCHECK^®^ from Sun Nuclear and MatriXX^®^ from IBA Dosimetry (GmbH, Germany). Another type of detector can be installed just below the collimator on the gantry head to measure the beam intensity distributions, e.g. COMPASS^®^ with MatriXX^®^ from IBA Dosimetry and DAVID^®^ from PTW (GmbH, Germany). Yet another technology in use for IMAT dosimetry is an electronic portal imaging detector (EPID), from which the images can be used to reconstruct the 3D dose distributions deposited in a patient during a treatment [6–8].

Over the last 15 years a further promising tool for 3D dose measurements has been developed that uses radiosensitive polymer gel dosimeters (RPGDs). A major advantage of the RPGD dosimetry is its unique ability to map true 3D distributions of the dose deposited in a uniform water-equivalent medium. This technique has been applied in clinically relevant dosimetric evaluations of radiation therapy plans of varying complexities, such as 3D conformal therapy, IMRT, stereotactic radiosurgery/radiotherapy, and brachytherapy [9]. Recently, Ceberg *et al.* used a 3D RPGD for verification of RapidArc^®^ plans [10]. They carried out an extensive evaluation of the RapidArc^®^ dose delivery using RPGD and Monte Carlo simulations. Concurrently, Watanabe *et al*. used a 3D RPGD for Tomotherapy^®^ treatment plans [11].

For this study we used BANG3 RPGD (MGS Research Inc., Madison, CT) to verify 3D dose distributions predicted by Eclipse™ treatment-planning software (Varian Medical Systems, Palo Alto, CA) for RapidArc^®^ treatment plans. The BANG3 results were compared with measurement results obtained by a gantry-mounted MapCHECK and Gafchromic^®^ EBT2 dosimetry films (Ashland Inc., Wayne, NJ).

## MATERIALS AND METHODS

### IMAT prostate cases

IMAT (or RapidArc^®^) plans were created for radiation therapy of two prostate cancer patients (patients R and E) by using the Eclipse™ treatment-planning system with the AAA algorithm, version 8.6.16 (Varian Medical Systems, Palo Alto, CA). The daily fraction size was 1.80 Gy for both plans. The treatments used a 10-MV photon beam from a Varian iX linear accelerator (Varian Medical Systems, Palo Alto, CA). A single full 360° arc with 45° collimator angle was used for these treatments. The planning target volumes (PTVs) of plans R and E were 233.6 cm^3^ and 164.2 cm^3^, respectively. The doses to the rectum were similar for the two plans, but the bladder dose for plan E was much smaller than that for plan R. The other major treatment planning parameters are summarized in Table 1. Note that an explicit model of the treatment couch was included in the treatment plans by assuming a pair of bars under the couch closed to minimize the attenuation of the photon beams by the thick bars.

**Table 1.** Treatment planning parameters for RapidArc prostate plans

Patient ID	R	E
Lateral separation (cm)	∼27.2	∼24.0
Photon energy	10 MV	10 MV
Number of fractions	8	5
Plan normalization value (%)	95.8	97.6
Number of arcs	1	1
Gantry rotation	179– 181° (CCW)	179– 181° (CCW)
Collimator angle	45.0°	45.3°
Total MU	822	632
PTV volume (cm^3^)	233.6	164.2
PTV min, mean, max doses (%)^a^	79.5, 104.4, 111.3	83.4, 102.6, 108.0
Rectum mean dose (%)^a^	46.0	37.4
Bladder mean dose (%)^a^	31.0	18.7
Right femoral head mean dose (%)^a^	30.0	28.2
Left femoral head mean dose (%)^a^	36.9	30.6

^a^100% = 1.8 Gy/fraction.

### Beam intensity distribution measurements

Treatment plans for patients R and E were evaluated for delivery accuracy using a 2D array detector MapCHECK^®^ (Sun Nuclear Corporation, Melbourne, FL). This device consists of an array of 445 diodes and covers an area of 22 cm × 22 cm. Before the IMAT measurements, it was calibrated by irradiating the device with a known dose of 100 cGy at an equivalent depth of 10 cm with a 10-MV photon beam (a 10 cm × 10 cm open field and 100 cm source-to-axis distance). For the dosimetric verification, the detector was placed in an isocentric mounting frame. Solid water phantoms were placed above the detector to create an equivalent depth of 10 cm tissue over the diodes. The verification plans were delivered to MapCHECK^®^ with the same method as for the patient's treatment (including the gantry rotation). The measured dose distributions were then compared with the calculations from Eclipse™ by using the absolute dose comparison mode on the MapCHECK software from the Sun Nuclear Co. The comparison was made using the gamma index with 3% dose difference and a 3 mm distance-to-agreement (DTA) criterion. All datapoints with doses > 10% of the normalization dose were included in the gamma analysis. It is noted that this measurement method produced a planar dose distribution integrated over the entire arc with a 10-cm build-up. Since the detector plane is always orthogonal to the beam axis, the issue of a diode detector being associated with oblique incident beams can be avoided.

### Film dosimetry

**Fig. 1.**
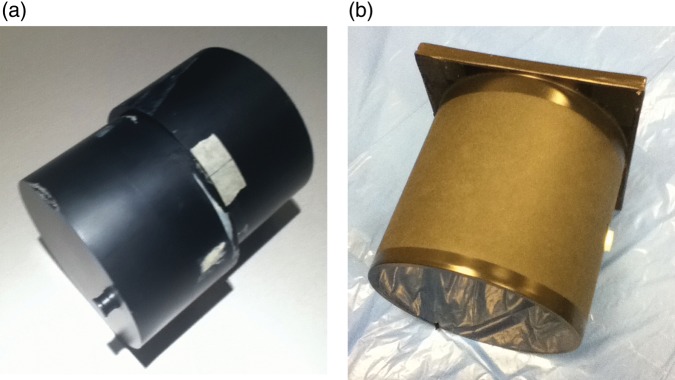
(**a**) Photo of cylindrical solid phantom used for film measurements. (**b**) Photo of BANG3 polymer gel phantom.

For film dosimetry we used a cylindrical acrylic phantom (16 cm diameter and 20 cm long), which was made of two parts as shown in Fig. 1a. The phantom was scanned with a CT scanner using 3-mm slice thickness. The CT images were used to create QA plans for patients R and E with Eclipse™. We used Gafchromic^®^ EBT2 dosimetry films (Ashland Inc., Covington, KY). For irradiation one sheet of film was cut to conform to the circular shape of the phantom cross section, and it was tightly placed between the two parts of the solid phantom on the treatment couch. The cylindrical axis of the phantom was perpendicular to the beam axis and in parallel to the gantry rotation axis at 100 cm from the radiation source. The films were read out by a flatbed scanner Model V700 (Epson–Seiko Corporation, Nagano, Japan) one day after the irradiation. The scanner was configured to scan the film with 150 dpi and 48-bit RGB. The scanned image was saved as a TIFF (Tagged Image File Format) image file.

The EBT2 films were calibrated by a field-by-field method with 13 dose-steps. An entire sheet of EBT2 film was divided into a 3 × 4 grid of 6 cm × 6 cm pieces in the central part of the sheet. The remaining pieces were used to obtain the optical density of unirradiated film. Each film piece was inserted into a solid-state water-equivalent phantom at the calibration depth of 10 cm. The films were irradiated with 0.25, 0.50, 0.75, 1.00, 1.25, 1.50, 1.75, 2.00, 2.50, 3.00, 3.50 and 4.00 Gy using a 10-MV photon beam. A characteristic curve was obtained using the signals for the red color channel because of its high sensitivity. We used the OmniPro-I'mRT software (IBA, Bartlett, IL) for film analyses. Quantitative assessment of the agreement between Eclipse™ and films was made using the gamma index with 3% dose difference and 3-mm DTA. The gamma analysis was done only for areas with a dose > 10% of the prescription dose.

### Polymer gel dosimetry

#### BANG3 polymer gel

We used a modified version of the BANG3 polymer gel dosimeter, BANG3-Pro-2 from MGS Research (Guilford, CT), which is currently only a commercial vendor of the RPGDs for radiation dosimetry [12]. The RPGD was contained in a 15-cm diameter and 15-cm long cylinder made of thin plastic walls (see Fig. 1b). Henceforth we call this a BANG3 phantom. The whole container was covered with black paper to prevent the sunlight damaging the RPGD. For dose–response calibration we used 10 small tubes (1 cm diameter and 8 cm long plastic tubes) filled with the BANG3 RPGD, (made at the same time as the gel used to fill the larger cylindrical phantom). The temperature environment where the RPGD is stored long term is an important factor affecting its gel response characteristics to radiation. Hence, we paid special attention to this issue by placing the BANG3 phantoms and calibration tubes in a refrigerator before usage, and in specific rooms to equilibrate the gel temperature with the room temperature during irradiation and MR scanning.

The calibration tubes were placed in a 37 cm × 37 cm × 37 cm water bath, Med Tec Small Water Tank Model MT 150 (Med Tec, Inc., currently CIVCO Medical solutions, Orange City, IA, USA) and irradiated with opposed parallel 10-MV photon beams. Using a 20 cm × 20 cm open field beam, a relatively uniform dose distribution was achieved throughout all of the calibration tubes. For the response calibration we applied 0.5, 1.0, 1.5, 2.0, 2.5, 3.0, 3.5 and 4 Gy to the tubes. A pair of un-irradiated calibration tubes was used as a control and attached to the surface of the BANG3 phantom for an MRI scan.

Two BANG3 phantoms were irradiated using dose delivery techniques identical to those used for the treatments of patients R and E. The phantoms were placed on the treatment couch in the same position as the solid phantom used for irradiation of the EBT2 films. Both CT-visible and MRI-visible markers were attached to the top, left side and right side of the cylindrical surface of the phantoms to indicate the machine isocenter location using the room lasers before irradiation.

One day after the irradiation, the BANG3 phantoms and the calibration tubes were scanned with a 3T MRI scanner, Magnetom Trio A Tim (Siemens Medical Solutions, Erlangen, Germany). The phantom was placed in a 12-channel head matrix coil by aligning the MRI-visible fiducial markers on the phantom with the in-room lasers for the scan. First, to determine the repetition time (TR), the spin-lattice relaxation time (T1) of the BANG3 RPGD was measured using a gradient echo technique. The measured T1 value of the RPGD was 1075 ms. Then we measured the spin–spin relaxation rate (R2) distribution by applying the multi-spin echo pulse sequence available on the Siemens MRI scanners (designated ‘cp_mc’ on the machine), which is a variation of the standard Car–Purcell–Meiboom–Gill (CPMG) pulse sequence. The imaging parameters were specifically selected for the RPGD as follows: the field-of-view = 256 mm × 256 mm, 256 × 256 pixels, 2-mm slice thickness without gap, TR = 7000 ms ( ∼ 7 × T1), echo spacing = 13.6 ms, and the number of echoes = 32. Each scan acquired 45 slices and took ∼ 90 min to complete. An interleaved slice acquisition method was chosen to minimize the interference between nearby imaging slices.

After irradiation and MRI scans, the BANG3 phantoms were scanned by a CT scanner with 3-mm slice thickness. Then, QA plans of patients R and E were created with Eclipse™. The beam isocenter was placed on the image using the CT-visible fiducial markers.

#### Analysis methods

The MRI data of the BANG3 phantoms and the calibration tubes were processed using an in-house MATLAB (The MathWorks, Inc., Natick, MA)-based program named ‘ATOM’. This program calculated the R2 values of all pixels from 32 images taken at 32 different echo times for all 45 slices (i.e. total 1440 slices.) It was assumed that the echo signal decays exponentially in time. The maximum likelihood estimation method was used to estimate the decay constant, i.e. R2, in the exponential decay equation. To improve the accuracy of the R2 estimation, the VAREC algorithm, which automatically selects the number of echo signals useful for the estimation, was used [13]. Note that the first echo signal was omitted from the R2 estimation.

The R2 values obtained from the calibration tubes were used to determine the correspondence between the dose absorbed in the RPGD and the R2 value. The final measured dose data were stored as a 256 × 256 × 45 3D matrix. Note that the voxel size for the measured dose distribution was 1 mm × 1 mm × 2 mm.

The dose distribution data calculated by Eclipse™ for the BANG3 phantoms were imported into the CERR program (Advanced Radiotherapy Treatment Planning Group, Washington University, St Louis, MO), with which we converted the dose data written in the Eclipse™-specific format into a standard matrix format readable by a MATLAB program. The calculated dose matrix sizes were 67 × 66 × 53 and 78 × 87 × 53 for plans R and E, respectively. The voxel size (or 3D grid size) of both calculation matrices was 2.5 mm × 2.5 mm × 3 mm. Note that the 3-mm grid size of the calculated dose in the *z*-direction was determined by the slice thickness used for the CT scan of the BANG3 phantoms.

3D dose comparison of the measured and calculated doses was accomplished using the in-house MATLAB-based programs PG2DCMP and PG3DCMP, which first recomputed the measured dose values at the 3D spatial grid-points of the computed dose matrix using a 3D linear interpolation algorithm. The program was then used to plot the isodose distributions on three orthogonal planes and the slice-by-slice isodose distributions, and to calculate the percentage dose differences as well as the gamma-index values. In particular we used dose-dependent dose difference (D4) diagrams, which can show the mean dose difference and the standard deviation at various dose levels.

The gamma-index was calculated using the PG3DCMP program. The details of the calculation algorithm have been published previously [11]. As the tolerances for the gamma-index calculation we chose 3% dose difference and 3-mm DTA. The relative dose difference in the gamma index formula was calculated as the difference between the measured dose and the calculated dose divided by a reference dose. For the current analyses we used 80% of the maximum dose as the reference dose (i.e. 2.32 Gy for plan R and 2.17 Gy for plan E). The threshold dose, above which the gamma analysis was performed, was varied to see its effect on the passing rate.

## RESULTS

### Beam intensity distribution measurements

The measurements of the MapCHECK detector yielded gamma passing rates of 99.3% and 97.1% for patients R and E, respectively. These results indicate that the delivered beam intensities of the two plans were in good agreement with the intended intensity distributions.

### Film dosimetry

**Fig. 2.**
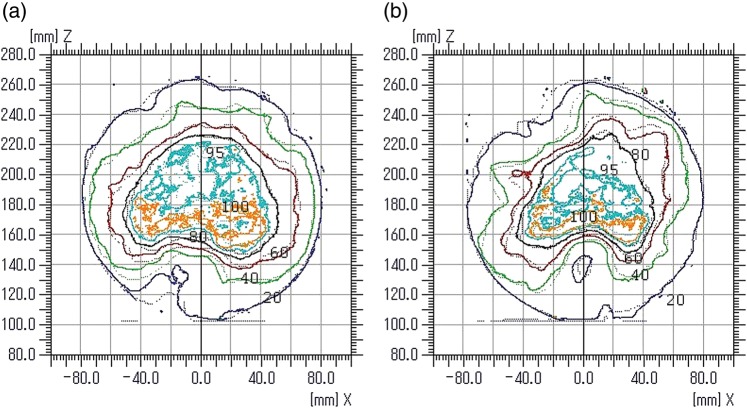
Overlays of isodose distributions from Gafchromic EBT2 film (solid line) and Eclipse calculation (dashed line) for patient R (**a**) and patient E (**b**). The 20, 40, 60, 80, 95 and 100% isodose lines are shown.

**Fig. 3.**
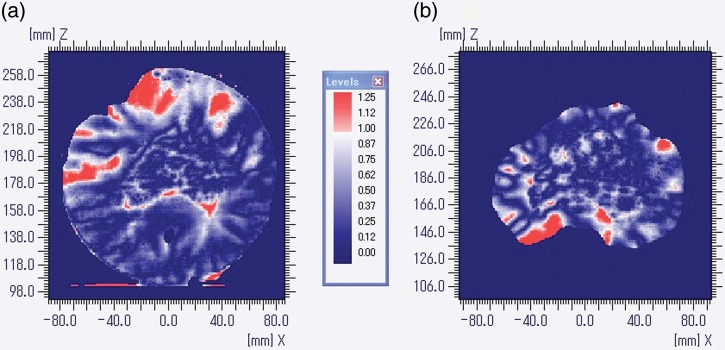
Gamma index maps for patient R (**a**) and patient E (**b**) corresponding to Fig. 2.

The isodose lines (20, 40, 60, 80, 95 and 100%) from the EBT2 film measurements and the Eclipse™ calculations are presented for patient R in Fig. 2a. A corresponding gamma index map is given in Fig. 3a. The gamma index values in some areas anterior to the PTV and the posterior edge of the PTV are greater than unity. The results for patient E are presented in Figs 2b and 3b. One can observe in Fig. 3b that there are areas with gamma index values greater than unity on the posterior side of the PTV, particularly near the edge of the film. A small gap between two pieces of the solid phantom may be enough to cause those large dose errors in the low-dose regions. The gamma passing rates were 96.5% and 92.6% for patients R and E, respectively.

### Polymer gel dosimetry

**Fig. 4.**
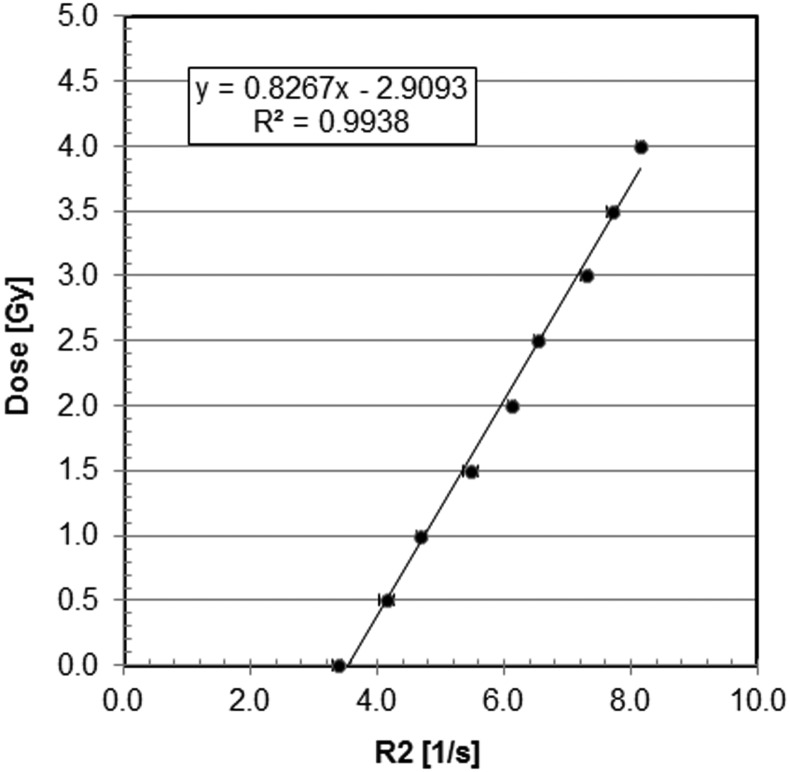
The relationship between dose and R2 for the BANG3 polymer gel dosimeter.

Analysis of calibration tubes gave us an R2-to-dose relationship for the BANG3 RPGD. Applying a regression method to the datapoints shown in Fig. 4, we obtained the following linear calibration equation for dose D [Gy] as a function of R2 [1/s]:

(1) $$D = 0.82 \cdot R2 - 2.90 $$

Here the coefficient of determination for the regression was 0.994, indicating fairly good linearity for the R2 and dose relationship. When the irradiated BANG3 phantoms were analyzed, however, the measured dose obtained directly from Eq. (1) showed about a factor of two higher doses than the calculations. Hence, the measured doses were normalized at a small region in the high-dose area inside the PTV to match them with the calculated doses.

**Fig. 5.**
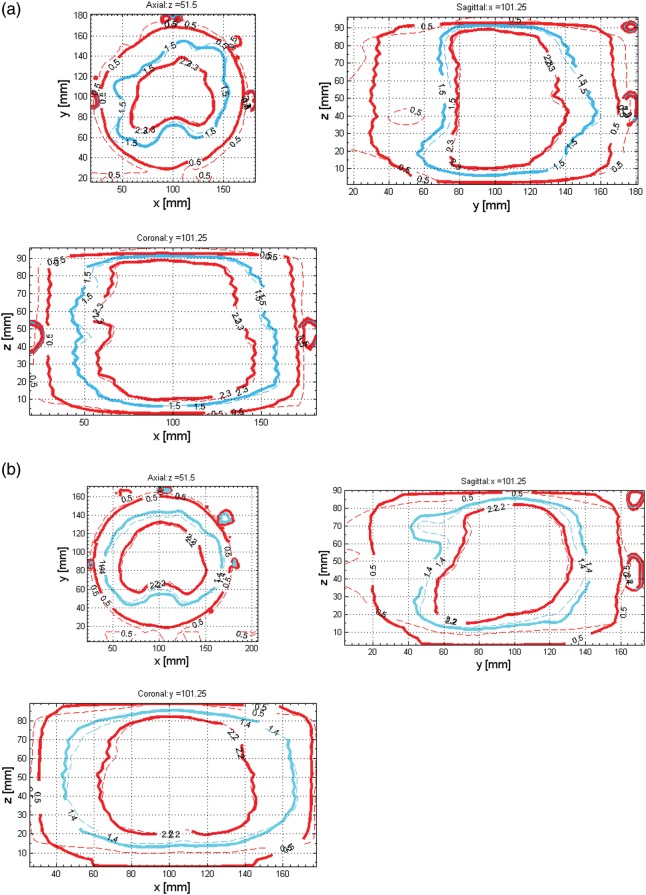
Isodose distributions for patient R (**a**) and patient E (**b**) from BANG3 measurement (thick lines) and Eclipse calculation (thin lines) plotted on three orthogonal planes that cross the machine isocenter. Three MRI-visible markers located on the top, left side and right side of the phantom surface were used to identify the isocenter location for the analyses.

Figure 5a and b show the isodose distributions for patients R and E, respectively. Isodose lines from BANG3 measurements and Eclipse™ calculations were plotted on three orthogonal planes, i.e. transverse, sagittal and coronal planes, that pass through the machine isocenter. Comparison of the 20%, 50% and 80% isodose lines from the measurements with the calculations indicate good agreement at higher dose regions for both cases.

**Fig. 6.**
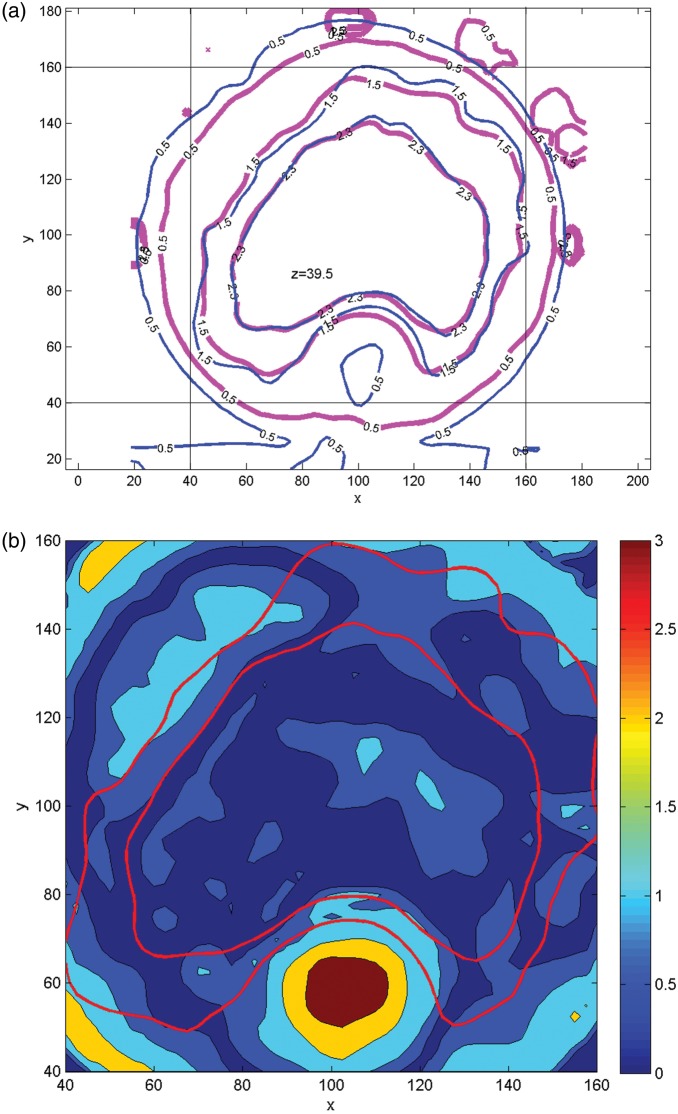
Comparison of BANG3 measured dose and calculated dose on a transverse plane (*z* = 39.5 mm) for patient R. (**a**) Isodose distributions: 20% (0.5 Gy), 50% (1.5 Gy) and 80% (2.3 Gy) isodose curves are shown. Red = BANG3 and Blue = Eclipse, (**b**) Gamma index maps: 80% isodose curves (red) are shown.

**Fig. 7.**
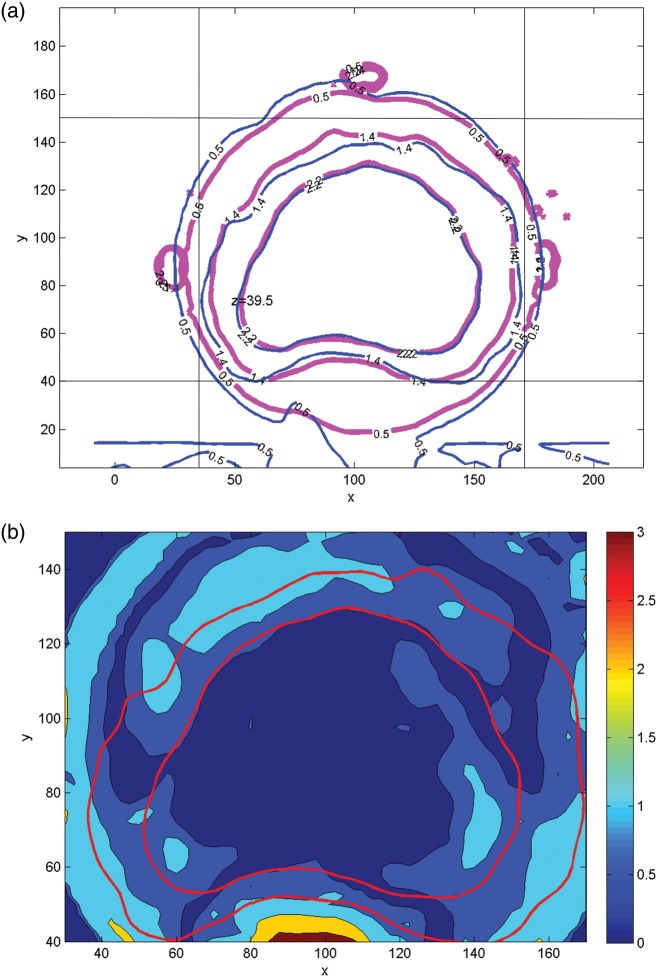
Comparison of BANG3 measured dose and calculated dose on a transverse plane (*z* = 39. 5 mm) for patient E. (**a**) Isodose distributions: 20% (0.5 Gy), 50% (1.4 Gy) and 80% (2.2 Gy) isodose curves are shown. Red = BANG3 and Blue = Eclipse. (**b**) Gamma index maps: 50% and 80% isodose curves (red) are shown. The color wash plot of the gamma index is shown for the region in the area surrounded by vertical and horizontal lines indicated in Fig.7 (**a**).

Figures 6a and 7a present the 20, 50 and 80% isodose lines of both the measured and calculated dose distributions on a transverse plane (*z* = 39.5 mm) along the axis of the cylindrical phantom, which corresponds to the superior–inferior direction in the accelerator coordinate system. Gamma index distributions for corresponding planes are shown in Figs 6b and 7b.

Figures 5, 6 and 7 indicate that the agreement between BANG3-measured and Eclipse™-calculated doses is excellent in higher dose regions, i.e. regions with doses > 80% of the maximum dose. However, the measured doses are lower than the calculations in lower dose regions, especially at below 50% dose level. This can be clearly seen in the area between the phantom surface and the 50% isodose lines (i.e. 1.5 Gy for plan R and 1.4 Gy for plan E) in the axial direction (Fig. 5a and b).

Figure 6a shows that there is a cold area on the posterior side of the PTV, i.e. at around *x* = 100 mm and *y* = 60 mm, which corresponds approximately to the location of the rectum. The Eclipse™ dose clearly shows a low-dose island, and the measurement also indicates a low dose in the same area, though the position and the size are slightly different from that of the Eclipse™ calculation. The corresponding Gamma map (Fig. 6b) shows that this area has a gamma value equal to or greater than unity, indicating poor agreement between the measured and calculated doses.

**Fig. 8.**
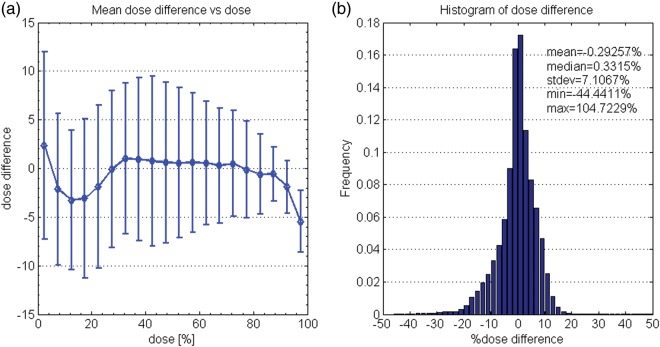
Comparison of BANG3 measured doses and calculated doses using a dose-dependent dose-difference diagram (**a**) and a percentage dose-difference histogram (**b**) for patient R.

**Fig. 9.**
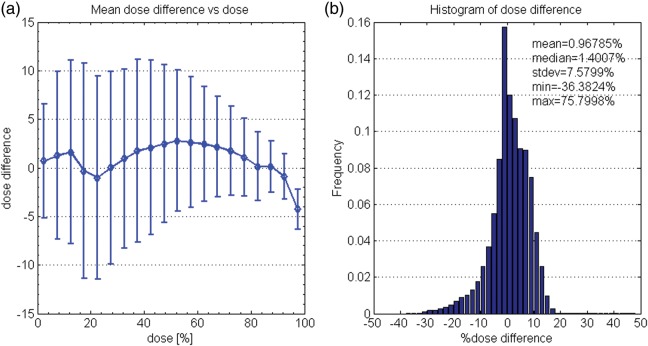
Comparison of BANG3 measured doses and calculated doses using a dose-dependent dose-difference diagram (**a**) and percentage dose-difference histogram (**b**) for patient E.

Dose-dependent dose-difference diagrams are presented in Fig 8a and 9a for patients R and E, respectively. As seen in Fig. 8a, the measured dose below 40% of the maximum dose is about 3% lower than the calculated dose for patient R. Figure 9a shows that agreement was better with patient E. Figures 8a and 9a also indicate that the mean dose differences for higher doses, i.e. for > 95% dose level, were about 5% for both cases. Figures 8b and 9b are histograms showing the percentage dose difference between the measured and calculated doses for patients R and E, respectively. Mean percentage differences between the measured and calculated doses in the 3D volumes were − 0.29 ± 7.1% and 0.97 ± 7.6% for patients R and E, respectively.

For a threshold dose of 80% of the maximum dose, the gamma passing rates were 93.7% and 92.9% for patients R and E, respectively. The gamma passing rates strongly depended on the threshold dose. The passing rates in a region covered by the 50% isodose surface (1.5 Gy for R and 1.4 Gy for E) decreased to 90.0% and 82.6% for patients R and E, respectively. This again indicates poor agreement of measured doses with calculated doses in the low-dose regions.

## DISCUSSION

We used three measurement methods to validate dose distributions of RapidArc^®^ plans. The beam intensity distribution measurements with an array detector, MapCHECK^®^, showed very good agreement between the measured and calculated distributions. The dose distributions obtained by Gafchromic EBT2 films agreed well with the calculations on a transverse plane orthogonal to the gantry rotation axis. Though the gamma passing rates obtained by the BANG3 measurements were lower than those obtained using the other measurements tools, the BANG3 results also showed a better gamma passing rate with patient R than patient E. Therefore, it is highly likely that the plan quality of patient R was better than that of patient E.

The results of RPGD dosimetry did not show very good agreement between measured and calculated dose distributions for the RapidArc^®^ plans in comparison with the published results [10, 14]. In fact, when lower dose regions in the measurement volume were included in the gamma analysis, the agreement observed in the current study was rather unsatisfactory. There are several reasons for the poor agreement. One reason is related to the characteristics of the RPGD dosimetry technique. Another cause is more to do with data processing.

In this study we used the RPGD as a relative dosimetry tool. To convert the R2 values to actual absorbed doses, we obtained a calibration relationship using small tubes filled with gel irradiated at eight different dose levels. The relationship obtained was represented by a linear equation. Because of the well-known difference in the radiation response of RPGDs contained in a small tube compared with a larger vessel [15], we need to rescale the measured dose to match the measured dose with the calculated dose. In the current study we normalized the measured dose in a small region inside the PTV. However, the true dose response for a specific RPGD could be different from the measured calibration data, even if the RPGDs were made in the same process. Both the slope and the intercept at the zero dose of the calibration equation can be different because of small but appreciable differences in the gel manufacturing conditions [16]. Furthermore, the relationship could be non-linear at higher doses due to saturation of polymer gel response. Hence, the rescaled measured dose tends to deviate from the calculated doses for the doses far from the normalization dose. The error is particularly large for doses much lower than the normalization dose [17]. These observations partially explain the poor agreement between the measured and calculated doses in regions with doses < 50% of the maximum dose.

Since the RPGD phantom was not sufficiently large relative to the size of the PTV for this study, ≥ 20% dose regions spread toward the edge of the BANG3 phantom. This led to a larger disagreement near the edge of the BANG3 phantom, where the dose response of the gel might be different from that in regions deep inside the container because of oxygen effects on the dose sensitivity and the non-uniform property of the gel in the regions close to the container surface. Hence, the gel container size must be sufficiently large in comparison with the size of the high-dose regions being measured for more accurate dosimetry (at the expense of the cost of the phantom material).

The slice thickness of MRI scans in the axial direction was 2 mm in contrast to the 1 mm × 1 mm pixel size of the transverse planes. Furthermore, the grid size used for the dose calculations with Eclipse™ was 2.5 mm × 2.5 mm × 3 mm. In particular, the grid size in the *z*-direction was limited by the slice thickness of the CT scans. This large grid size in the *z*-direction, hence, caused large differences at the superior and inferior ends of the PTV, where the dose gradients were large.

One of the justifications for using a true 3D dosimetry technique such as RPGD is its potential to evaluate the dose to critical structures, or organs-at-risk (OARs), in comparison with the dose predicted by the treatment-planning software. For the current study, one of the OARs was the rectum, located just posterior to the prostate. The RapidArc^®^ plans were created to spare the rectum, leading to a highly non-uniform dose volume around the rectum. Evaluation of the actually delivered dose to the rectum, hence, is clinically very important. Since we could not directly calculate the dose–volume histogram of the rectum using the current version of our software, we could only visually inspect the dose difference in and around the rectum. This goal was only partially achieved by this study using BANG3 because of the poor accuracy of the measured dose in the low-dose range.

It should be noted that, despite some shortcomings, measurements using RPGD provide true 3D dose distributions, which cannot be obtained by any other existing methods. Upon introduction of the IMAT technology to the clinical setting, several dosimetric tools were specifically developed for so-called ‘3D’ dosimetry. Those devices include ArcCheck^®^, Delta4^®^, EPID, MapCHECK^®^, COMPASS^®^/MatriXX^®^, etc. Despite the manufacturers' or the developers' claims, none of these technologies can provide us with ‘true’ 3D dose measurement capability because they involve some form of interpolation/reconstruction/mapping of 2D/semi-2D data into 3D space. Hence, polymer gel dosimetry will continue to have its place within the wide range of available tools.

## CONCLUSIONS

This study has shown that IMAT prostate plans can be delivered with high accuracy. This conclusion was supported by three independent dose measurements for two prostate cases. We used three dosimetric tools that vary in their dose measurement capability: a MapCHECK^®^ diode array detector, radiochromic EBT2 films, and BANG3 RPGD. Our comparison indicated that MapCHECK^®^ fluence measurement is too optimistic and may miss the fine details of the RapidArc delivery problem. The precision of the BANG3 RPGD measurement still needs further improvement if it is to be used as a sole QA tool, despite the enormous amount of information contained in its data. Currently, the EBT2 film measurement is the most practical tool in terms of precision, ease of use, and cost.

The 3D measurements with the BANG3 RPGD showed less satisfactory agreement with the treatment plans. Agreement of 3D evaluation between the measured and the calculated doses was not excellent, in particular, in low-dose regions outside the target volume. Hence, further improvement of the RPGD dosimetry technique is warranted, with special attention to more accurate measurements in low-dose regions. As potential causes for the lack of agreement we identified the low quality of the dose-to-R2 calibration data, and the size of the phantom not being sufficiently large relative to the irradiated volume of interest. Provided these issues are resolved, RPGD dosimetry will also be applied to accurate measurement of the dose to non-PTV regions including OARs. 3D evaluation will increase its value when a treatment with even higher doses is attempted and more strict dose tolerance criteria for OARs are used in the future.

## FUNDING

We were partially supported by Grant-in-Aid for Young Scientists (B) (KAKENHI: Grant number: 22791227). MRI scans for this study were performed at the Center for Magnetic Resonance Research (CMRR), and was partially funded by NIH grants in USA (P30 NS057091, P41 RR008079).
